# Global target mRNA specification and regulation by the RNA-binding protein ZFP36

**DOI:** 10.1186/gb-2014-15-1-r12

**Published:** 2014-01-08

**Authors:** Neelanjan Mukherjee, Nicholas C Jacobs, Markus Hafner, Elizabeth A Kennington, Jeffrey D Nusbaum, Thomas Tuschl, Perry J Blackshear, Uwe Ohler

**Affiliations:** 1 Berlin Institute for Medical Systems Biology, Max Delbrück Center for Molecular Medicine, Robert-Rössle-Strasse 10, 13125 Berlin, Germany; 2 Laboratory of Signal Transduction, National Institute of Environmental Health Sciences, Research Triangle Park, NC 27709 USA; 3 Howard Hughes Medical Institute and Laboratory for RNA Molecular Biology, The Rockefeller University, New York, NY 10065 USA

## Abstract

**Background:**

ZFP36, also known as tristetraprolin or TTP, and ELAVL1, also known as HuR, are two disease-relevant RNA-binding proteins (RBPs) that both interact with AU-rich sequences but have antagonistic roles. While ELAVL1 binding has been profiled in several studies, the precise *in vivo* binding specificity of ZFP36 has not been investigated on a global scale. We determined ZFP36 binding preferences using cross-linking and immunoprecipitation in human embryonic kidney cells, and examined the combinatorial regulation of AU-rich elements by ZFP36 and ELAVL1.

**Results:**

Targets bound and negatively regulated by ZFP36 include transcripts encoding proteins necessary for immune function and cancer, and transcripts encoding other RBPs. Using partial correlation analysis, we were able to quantify the association between ZFP36 binding sites and differential target RNA abundance upon ZFP36 overexpression independent of effects from confounding features. Genes with increased mRNA half-lives in ZFP36 knockout versus wild-type mouse cells were significantly enriched for our human ZFP36 targets. We identified thousands of overlapping ZFP36 and ELAVL1 binding sites, in 1,313 genes, and found that ZFP36 degrades transcripts through specific AU-rich sequences, representing a subset of the U-rich sequences ELAVL1 interacts with to stabilize transcripts.

**Conclusions:**

ZFP36-RNA target specificities *in vivo* are quantitatively similar to previously reported *in vitro* binding affinities. ZFP36 and ELAVL1 bind an overlapping spectrum of RNA sequences, yet with differential relative preferences that dictate combinatorial regulatory potential. Our findings and methodology delineate an approach to unravel *in vivo* combinatorial regulation by RNA-binding proteins.

## Background

Regulation of gene expression is a complex process coordinated at many steps. Post-transcriptional regulation is controlled through RNA-binding proteins (RBPs) and non-coding RNAs (ncRNAs) interacting with RNA regulatory elements (RREs). Dynamic and combinatorial interactions of RBPs and ncRNAs with these RREs determine the functional outcome of specific steps of RNA processing, such as splicing, polyadenylation, export, stability and translation [1]. Additionally, interactions between RBPs and RREs govern messenger RNA (mRNA) stability and translational efficiency.

AU-rich elements (AREs) are conserved *cis*-regulatory elements, originally discovered in the 3′ untranslated region (UTR) of labile mRNAs, encoding cytokines and early expressed immune response genes [2,3]. The most commonly defined AREs are the pentamer AUUUA, which is often found tandemly arranged, and the nonamer UUAUUUAUU, which is a pentamer flanked by uridylates [4-6]. AREs are generally considered instability elements, since they typically interact with RBPs that subsequently recruit mRNA degradation machinery [7,8]. Examples of ARE-binding proteins include the ELAV family, ZPF36 family, HNRNPD/AUF family, TIA1, TIAR and KSRP. Mice lacking the AU-rich region containing multiple AREs residing in the 3′ UTR of the tumor necrosis factor α (TNFα) transcript develop inflammatory disease, demonstrating their primacy in gene regulatory mechanisms [9].

Tristetraprolin (ZFP36) is the founding member of the TIS11 family of RBPs. It interacts with AREs through tandem CCCH zinc fingers (TZFs) [10]. Mice deficient in ZFP36 also exhibit inflammatory disease phenotypes, mostly explained by deregulation of TNFα mRNA stability and biosynthesis [11]. The interaction of ZFP36 with AREs in the 3′ UTR of targeted mRNAs, such as TNFα, promotes mRNA degradation [12]. ZFP36 is a nucleo-cytoplasmic shuttling protein that is predominantly cytoplasmic at steady state [13]. The RNA-binding specificity of ZFP36 resides in the TZF domain, which interact with low nanomolar affinity with synthetic RNA substrates containing the nonamer UUAUUUAUU [14]. The NMR structure of the nonamer sequence bound to the TZF domain from human ZFP36L2 revealed that the zinc fingers symmetrically bind to UAUU half-sites, with the 5′ uridylate unbound [15]. There have been many detailed *in vitro* investigations of ZFP36–mRNA interaction (reviewed in [16]); however, *in vivo* studies have only determined the mRNA pools associated with ZFP36 and have not defined individual binding sites at high resolution [17,18].

Although many ARE-binding RBPs recruit mRNA degradation complexes, the ELAV/Hu family of proteins binds to AREs and promotes mRNA stability and translation [2,3,19-21]. Both *in vitro* and *in vivo* studies have determined that Hu proteins interact with AREs, as well as U and CU-rich sequences [4-6,22,23]. These sequence preferences were substantiated *in vivo* using photoactivatable ribonucleoside cross-linking and immunoprecipitation (PAR-CLIP) to define high-resolution interaction sites for the ubiquitously expressed family member ELAVL1 [7,8,24-26]. Due to T-to-C transitions indicative of RBP–RNA interaction sites [10,27], PAR-CLIP gives a higher resolution of binding sites than earlier methods [11,28,29]. A commonly postulated mechanism through which ELAVL1 promotes mRNA stability is competition with ARE-binding RBPs that promote degradation. Like ZFP36, ELAVL1 is a nucleo-cytoplasmic shuttling protein, but under normal conditions is predominantly nuclear localized [12,30], consistent with numerous intronic binding sites found in PAR-CLIP experiments [13,24-26]. Simultaneous temporal and spatial competition for binding sites by ELAVL1 and ZFP36 is important when responding to stimuli, particularly immune activation, upon which ELAVL1 redistributes from the nucleus to the cytoplasm [14,31] and ZFP36 is induced and cytoplasmically localized. There is evidence for competitive binding and displacement of ZFP36 with increasing amounts of ELAVL1 and/or MAPK signaling influencing the combinatorial regulation of the TNFα mRNA by ELAVL1 and ZFP36 [15,32]. However, the extent to which similar competitive mechanisms exist transcriptome-wide is unknown.

Conflated direct and indirect relations are a concern, particularly when integrating multiple genomic measurements to understand complex gene regulatory mechanisms. An example of a relation relevant for post-transcriptional regulation is that longer transcripts contain more binding sites for *trans*-acting factors. Consequently, significant correlations between 3′ UTR length and miRNA-dependent downregulation have been found in numerous datasets [16,33]. Overexpression of ZFP36 in the absence of stress has been demonstrated to promote stress granule assembly [18], potentially causing non-ZFP36 specific effects in transcript abundance. Partial correlation analysis quantifies the direct dependence between two variables (for example, number of binding sites or expression changes) when accounting for other variables (for example, UTR length and mRNA abundance). It has recently been applied to investigate genome evolution [34,35], as well as chromatin state and transcriptional regulation [36]. Here we apply it to account for generic transcript features when inferring functional relations between the number of binding sites and gene expression changes due to perturbing the expression of the RBP.

There are numerous examples of combinatorial binding of RBPs resulting in specific splicing decisions [37-40], but this has not been as extensively studied for mRNA stability. Given the numerous ARE-binding RBPs, the principles underlying the integration of RBP binding and concomitant regulatory outcomes have remained elusive. We therefore determined the *in vivo* mRNA-binding sites of ZFP36. The interaction sites and sequence preferences were compared to similarly acquired *in vivo* HuR interaction data, specifically contrasting AU-rich sequences with predominantly U- and CU-rich sequences. Our results indicate active competition between ZFP36 and ELAVL1 for commonly used AU-rich sequences and define the characteristics of these combinatorial regulatory events.

## Results and discussion

### ZFP36 preferentially binds to 3′ UTRs of mRNAs encoding regulators of gene expression

**Figure 1:**
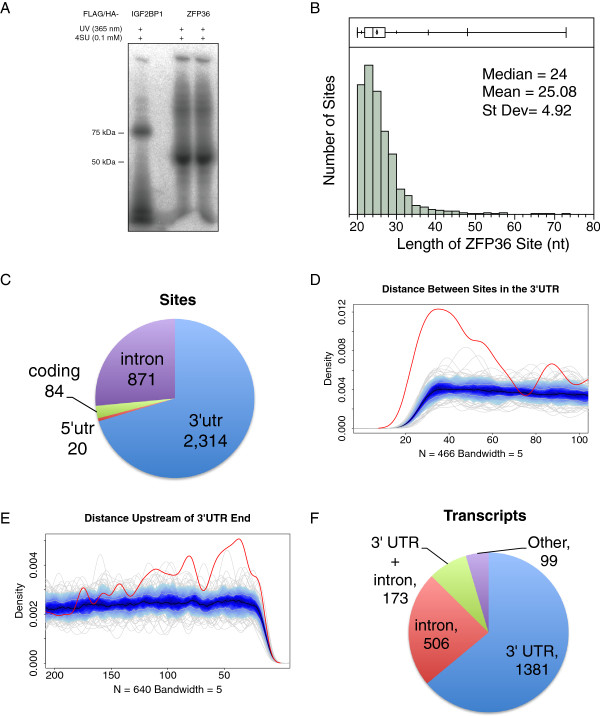
**ZFP36 RNA binding site characteristics. (A)** Phosphor image of the SDS-PAGE fractionating of the PAR-CLIP immunoprecipitate from doxycycline-induced FLAG/HA-tagged ZFP36 and IGF2BP1 (specificity control) HEK293 cells. **(B)** Length distribution of ZFP36 mRNA binding sites. **(C)** Proportion of binding sites mapping to different mRNA regions. **(D)** ZFP36 site distribution in 3′ UTRs (red line). Median (black), interquartile range (dark blue), interdecile range (light blue) and the outliers (grey lines) are shown for the background. **(E)** Over-representation of ZFP36 sites (red line) at the distal end of 3′ UTRs relative to the background. **(F)** Proportion of genes with specific regions of ZFP36 binding sites. nt, nucleotide; PAR-CLIP, photoactivatable ribonucleoside cross-linking and immunoprecipitation; St Dev, standard deviation; UTR, untranslated region; SDS-PAGE, sodium dodecyl sulfate polyacrylamide gel electrophoresis.

We employed PAR-CLIP to identify the *in vivo* ZFP36 binding sites transcriptome-wide in human embryonic kidney cells (HEK293) [41]. We chose HEK293 cells and FLAG/HA-tagged ZFP36 to be as consistent as possible with previous relevant PAR-CLIP experiments with other RBPs, specifically ELAVL1. Ribonuclease-treated, immunoprecipitated and radiolabeled ribonucleoprotein (RNP) complexes resolved by sodium dodecyl sulfate polyacrylamide gel electrophoresis (SDS-PAGE) revealed a major band at approximately 50 kDa corresponding to the FLAG/HA-tagged ZFP36 (Figure 1A). The cDNA sequence reads were processed and aligned to the human genome (see Materials and methods and Additional file 1 for detailed parameters). PARalyzer, an algorithm that utilizes PAR-CLIP T-to-C transitions indicative of RBP–RNA interactions, distinguished 4,626 clusters genome-wide (also referred to as binding sites, Additional file 2: Table S1) with a mean length of 25 nucleotides from 328,433 uniquely aligned T-to-C reconciled reads (Figure 1B and Additional file 3: Figure S1A) [42]. Of the binding sites mapping to annotated and repeat-masked sequences (3,497), those located in mRNA accounted for 94% (3,289).

The majority of ZFP36 mRNA binding sites (70%, 2,314) mapped to the 3′ UTR, and very few binding sites were located at the 5′ UTR or the protein coding region (Figure 1C). Approximately one-quarter of the mRNA binding sites were intronic (871), which is conceivable since ZFP36 is a known nucleo-cytoplasmic shuttling RBP [13]. However, the 3′ UTR sites had a substantially higher T-to-C conversion fraction and specificity relative to ZFP36 sites from other annotation categories including introns, 5′ UTRs and protein coding regions (Additional file 3: Figure S1B). We observed closely spaced ZFP36 sites within 3′ UTRs (Figure 1D), indicative of multimerization of ZFP36 and supported by results from 2-hybrid analysis and immunoreactive bands migrating at twice the expected number of kilodaltons of ZFP36 in cell extracts (PJB, unpublished data). Of the 1,148 transcripts with more than one ZFP36 site in the 3′ UTR, 228 had two sites within 70 nucleotides, the range for which proximity of ZFP36 sites is above background. The statistically significant enrichment for ZFP36 sites in the last 100 nucleotides of the 3′ UTR (Figure 1E) was intriguing given its ability to promote deadenylation [43]. This spatial bias was not observed for the sites of all other investigated 3′ UTR binding RBPs (Additional file 3: Figure S1D) and thus not due to biases in library generation, though it may be related to AU-richness proximal to polyadenylation sites [44]. Of the 2,143 genes with ZFP36 sites, 64% exclusively had 3′ UTR sites, 23% exclusively had intronic sites and only 8% contained both 3′ UTR and intronic sites (Figure 1F). Genes with 3′ UTR sites were less likely to also have intronic sites in contrast to ELAVL1 [25]. The strong preference for 3′ UTR binding was anticipated for an RBP predominantly involved in cytoplasmic regulation of mRNA stability and translation.

**Figure 2:**
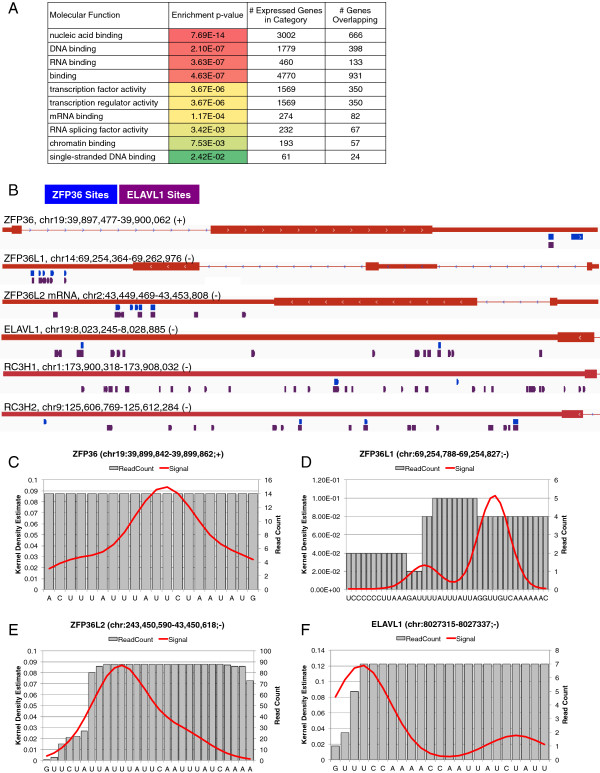
**ZFP36 binds to mRNAs encoding regulatory proteins. (A)** Gene ontology enrichment analysis of genes with ZFP36 binding sites ranked and shaded from red (lowest) to green (highest) by Bonferroni corrected *P* values (< 0.05) from Panther DB. **(B)** Distribution of ZFP36 binding sites (red) and ELAVL1 binding sites (purple) from PAR-CLIP data for immune-relevant RBPs. The genomic span is indicated for each gene and these spans vary greatly. **(C-F)** Visualization of individual ZFP36 binding sites with the T-to-C conversion density estimate (red line) and read counts (grey) within the ZFP36, ZFP36L1, ZFP36L2 and ELAVL1 3′ UTRs. chr, chromosome; PAR-CLIP, photoactivatable ribonucleoside cross-linking and immunoprecipitation; RBP, RNA-binding protein; UTR, untranslated region.

ZFP36 sites were found to be significantly enriched in transcripts encoding transcriptional and post-transcriptional regulatory proteins (Figure 2A). In addition to its own 3′ UTR (Figure 2B,C), we detected ZFP36 binding to numerous sites within the 3′ UTR of several other RBPs necessary for proper immune function [11,45-47], including ZFP36 family members ZFP36L1 and ZFP36L2 (five sites each; Figure 2B,D,E), ELAVL1 (two sites; Figure 2B,F), as well as RC3H1 and RC3H2 (two and three sites, respectively; Figure 2B). We also found that most of the ZFP36 sites in these 3′ UTRs overlapped with ELAVL1 sites (Figure 2B). The similarity of the binding domain and sequence specificity of other ZFP36 family members and the presence of both ZFP36 and ELAVL1 binding sites in these 3′ UTRs warrant future investigation as a network motif that could potentially produce interesting temporal expression patterns, particularly in the context of stimuli inducing ZFP36 protein expression.

### *In vivo* ZFP36 binding specificity

**Figure 3:**
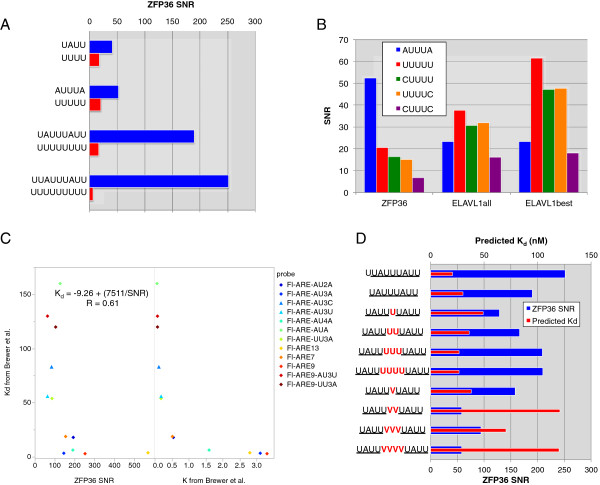
***In vivo *ZFP36 binding specificity. (A)** SNR values for specific motif occurrences within ZFP36 3′ UTR sites relative to the background. **(B)** Comparison of SNR values for indicated pentamer sequences in ZFP36 sites, all ELAVL1 sites (ELAVL1_all_), as well as the highest read count (ELAVL1_best_) subset of all ELAVL1 sites. **(C)** Comparison of SNR values with *K* and *K*_*d*_ of probe sequences (in different colors) determined using fluorescence anisotropy experiments [48]. The correlation coefficient and equation for the fit of *K*_*d*_ to the reciprocal of the SNR is indicated. **(D)** Comparison of SNR values (blue) and predicted *K*_*d*_ (red) of nonamer and octamer sequences, as well as half-sites interrupted by one to four uridylates (U) or a non-uridylate (V = A/C/G). SNR, signal-to-noise ratio.

Since *de novo* motif finding in PARalyzer-defined ZFP36 binding sites confirmed AU-rich sequences as the most highly enriched sequence motifs (data not shown), we utilized the wealth of detailed *in vitro* experimental evidence for ZFP36 RNA substrate specificity (reviewed in [16]) to guide more detailed analyses. More than 84% of the mRNA sites contained known ZFP36 RREs: the classic nonamer UUAUUUAUU (8%), the octamer UAUUUAUU (14%) identified as the residues contacted by the TZF domain in the TIS11D structure, a single UAUU half-site (82%), or the AUUUA pentamer (47%). A similar fraction of intronic ZFP36 sites (804 of 871) contained one or more of the above RREs, indicating their potential for functional relevance. To assess the enrichment of each individual RRE we calculated a signal-to-noise ratio (SNR). This reflects the rate of occurrence of a specific sequence in ZFP36 sites relative to the expected rate of occurrence in the background sequence, in this case within 3′ UTRs (see Materials and methods for details). Each of the above RREs were found 40 to 250 times more often in the ZFP36 sites than expected by chance and were at least twice as enriched as polyU-stretches of the same respective length (Figure 3A). Since the ELAVL1 library was sequenced at higher depth than the ZFP36 library, we used a size-matched subset of the ELAVL1 library using the top sites by read count (ELAVL1_best_) to compare SNR values of various U-rich pentamer sequences in the ZFP36 to ELAVL1 PAR-CLIP data (Additional file 3: Figure S1C). The AUUUA pentamer was relatively more enriched in the ZFP36 sites, whereas UUUUU and U-stretches flanked by C on both or either end were relatively more enriched in ELAVL1 sites (Figure 3B). These contrasting sequence preferences are consistent with highly quantitative *in vitro* binding studies of both proteins, which also found that ZFP36 binds with higher affinity to AU-rich sequences relative to U-rich and CU-rich sequences, and vice versa for ELAVL1 [48,49].

Previously reported *in vitro* ZFP36 binding studies quantified the binding of numerous RNA probes to the 73 amino acid portion of ZFP36 containing the TZF using fluorescence anisotropy measurements [48]. For each probe, we compared the experimentally determined association, *K*, and disassociation constant, *K*_*d*_, with the SNR values calculated from our ZFP36 PAR-CLIP data matching experimental RNA probe and *in vivo* binding site sequences. We observed a very strong correlation with *K*_*d*_ values (Figure 3C). This strong concordance allowed us to calculate predicted *K*_*d*_ values from the PAR-CLIP derived SNR ratios and thus perform *in silico* experiments with specific sequences. The relation between SNR and *K*_*d*_ was non-linear. For example, larger differences between the SNR values for the nonamer (UUAUUUAUU) and the octamer (UUAUUUAU) translated to smaller differences in the predicted *K*_*d*_ values for high affinity targets (Figure 3D). When inserting a spacer in the octamer between the UUAU half-sites bound by the TZF domain, a single nucleotide disruption by any nucleotide resulted in similar increases in predicted *K*_*d*_ values. However, multiple continuous disruptions with non-uridylates exhibit quite large increases in predicted *K*_*d*_ values, whereas disruptions with uridylates had little to no change (Figure 3D). These results highlight the quality and complementary value of the PAR-CLIP method, as well as the detailed *in vitro* biochemical analysis of ZFP36 substrate specificity.

### Specificity of ZFP36 function

To evaluate the regulatory function of ZFP36, we used microarray analysis to compare transcript abundance before and after doxycycline-induction of EGFP-ZFP36 fusion protein expression in HEK293 cells otherwise expressing little endogenous ZFP36 (Additional file 4: Figure S2A,B and Additional file 5: Table S2). We detected 2,784 genes with statistically significant expression differences by comparing overnight doxycycline-induced cells to mock-induced cells. Doxycycline treatment of the parental cell line did not yield any statistically significant differences defined by Bonferroni corrected *P* < 0.01 (Additional file 4: Figure S2C). Only 45% of the differentially expressed genes decreased as a result of ZFP36 overexpression (downregulated genes). The large number of upregulated genes was likely due to secondary effects from prolonged ZFP36 overexpression (Additional file 4: Figure S2D). (Detailed characterization of genes with ZFP36 and ELAVL1 sites with numerous other motif contributions for significantly upregulated and downregulated genes are shown in Additional file 4: Figure S2H.) The 1,254 significantly downregulated genes decreased by approximately 3.5 fold on average. The breadth and degree of expression differences resulting from EGFP-ZFP36 overexpression were distinctly greater than the typical fine-tuning associated with most RBPs and miRNAs. This is particularly striking in comparison to ELAVL1 where only 15 genes exhibited greater than a twofold change due to ELAVL1 knockdown (Additional file 4: Figure S2G from [25]).

Downregulated genes were significantly enriched for AU-rich sequences in an unbiased analysis of the contribution of 3′ UTR motif elements to expression changes (Additional file 4: Figure S2E) [50]. Thus, we examined the association between the number of counts per 3′ UTR of specific AREs and EGFP-ZFP36-induced changes in mRNA abundance. The RREs described by the ZFP36L2:ARE NMR structure, the UAUUUAUU octamer and the UAUU half-site, had the strongest association with ZFP36-induced changes in mRNA abundance. The UAUUUAUU octamer had a stronger association with ZFP36-induced changes in mRNA abundance than the UUAUUUAUU nonamer (Additional file 4: Figure S2F). The UAUU half-site had a stronger association with ZFP36-induced changes in mRNA abundance than the AUUUA pentamer (Additional file 4: Figure S2F).

**Figure 4:**
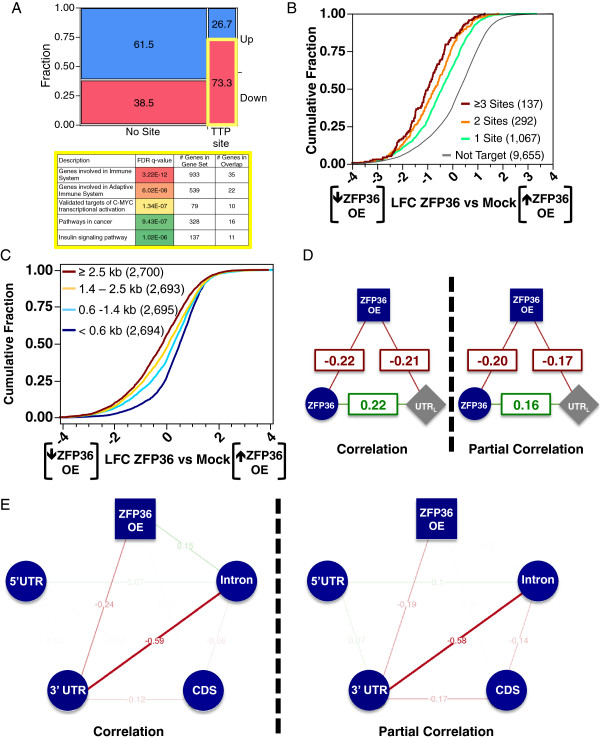
**Functional sensitivity of ZFP36 sites. (A)** Mosaic plot of significantly differentially upregulated (blue) and downregulated genes (pink) with and without ZFP36 sites. Significantly enriched MSigDB canonical pathway gene sets for downregulated genes with ZFP36 sites (yellow outline). Cumulative distribution function plots of EGFP-ZFP36-induced changes in mRNA abundance for 3′ UTRs grouped **(B)** by number of ZFP36 sites and **(C)** by 3′ UTR length. For all correlation plots the color and shape of the node indicates specific data classes (circles: PAR-CLIP sites, squares: expression change upon RBP perturbation, grey diamonds: transcript features, blue: ZFP36-related feature, purple: ELAVL1-related feature). Edge line thicknesses are proportional to correlation coefficient strength and the direction of correlation is indicated by the sign and color (negative in red and positive in green). **(D)** Correlation (left) and partial correlation (right) analysis of number of ZFP36 sites per 3′ UTR (blue circles), EGFP-ZFP36-induced changes in mRNA abundance (blue squares) and 3′ UTR length for all genes. **(E)** Correlation (left) and partial correlation (right) analysis of the number of ZFP36 sites per mRNA region and EGFP-ZFP36-induced changes in mRNA abundance for all genes with at least one ZFP36 site. kb, kilobase; PAR-CLIP, photoactivatable ribonucleoside cross-linking and immunoprecipitation; TTP, tristetraprolin; UTR, untranslated region; FDR, false discovery rate; LFC, log_2_ fold change; OE, overexpression; CDS, coding sequence.

Among differentially expressed genes, those containing ZFP36 sites were twice as often downregulated compared to those not containing ZFP36 sites (Figure 4A). Pathway enrichment analysis showed that downregulated genes with ZFP36 sites were enriched in immune and insulin signaling genes (Figure 4A). This is consistent with early studies demonstrating ZFP36-dependent immune system phenotypes as well as rapid insulin-stimulated ZFP36 induction [10,11]. Recent studies have identified that ZFP36 acts as a tumor suppressor in Myc-induced lymphomas, in which Myc directly binds to an initiator element downstream of the TATA box to suppress ZFP36 transcription. The resulting deregulation of numerous ARE-containing genes contributes to the development and maintenance of the malignant state, which can be abolished by restoring ZFP36 [51]. The top five significantly enriched pathways were remarkably accurate for the known physiological roles of ZFP36 and were not enriched for ZFP36 target genes without significant changes in expression.

To illustrate functional effects, genes are frequently stratified by a variable of interest (such as the presence or absence or number of binding sites), followed by a statistical test for significant phenotypic differences (such as expression changes) between the subsets. For instance, we observed strong associations between the number of ZFP36 sites in the 3′ UTR of a gene and its downregulation due to EGFP-ZFP36 overexpression (Figure 4B), between 3′ UTR length and EGFP-ZFP36-induced changes in mRNA abundance (Figure 4C), and between ZFP36 sites and 3′ UTR length. These effects were corroborated by the strength of Spearman correlations between all three variables (Figure 4D, left). However, pairwise correlations are not able to quantify direct and indirect relations for more than two variables and thus we employed partial correlation analysis. Comparing the correlation and partial correlation of ZFP36 sites and 3′ UTR length with EGFP-ZFP36-induced changes in mRNA abundance, most of the relation between ZFP36 sites and EGFP-ZFP36-induced changes in mRNA abundance was independent of 3′ UTR length and vice versa (Figure 4D). Secondary effects from prolonged ZFP36 overexpression, such as spontaneous stress granule assembly [52] and changes in the expression levels of other RBPs, were potential explanations for the independent contribution of 3′ UTR length to EGFP-ZFP36-induced changes in mRNA abundance.

We determined which transcript regions contained the most independent information for explaining EGFP-ZFP36-induced changes in mRNA abundance, without having to lose statistical power by separating transcripts into mutually exclusive categories, such as binding within introns only or 3′ UTRs only. EGFP-ZFP36-induced changes in mRNA abundance correlated negatively with the number of ZFP36 3′ UTR sites and positively with the number of intronic ZFP36 sites (Figure 4E, left). Partial correlation analysis revealed that ZFP36 sites outside of 3′UTRs, including introns, did not contribute any additional independent explanation of EGFP-ZFP36-induced changes in mRNA abundance (Figure 4E, right). Here, the strong negative association or avoidance between the number of intronic and 3′ UTR sites per gene (like the results in Figure 1F) acts as a mediator in explaining the spurious effects between intronic ZFP36 sites and EGFP-ZFP36-induced changes in mRNA abundance. This result does not rule out that ZFP36 sites from other transcript regions have other functional effects, but we only utilized 3′ UTR binding sites for further analysis.

We compared our ZFP36 PAR-CLIP and overexpression data from human HEK293 cells with global mRNA stability changes after serum stimulation for ZFP36 wild-type (WT) or knockout (KO) mouse fibroblast cell lines [53]. Genes with increased mRNA half-lives in the ZFP36 KO vs WT mouse cells were significantly enriched for genes with ZFP36 PAR-CLIP sites in 3′ UTR (*P* = 0.003) and genes significantly downregulated upon EGFP-ZFP36 overexpression (*P* = 0.001), but were not significantly enriched for genes significantly upregulated upon EGFP-ZFP36 overexpression (*P* = 0.1) (Additional file 6: Figure S3A). Interestingly, ELAVL1 RIP-chip targets from human Jurkat T-cell activation were significantly enriched in genes with increased mRNA half-lives in the ZFP36 KO vs WT mouse cells, even though ZFP36 RIP-chip targets from activated mouse macrophages were slightly but not significantly enriched [18]. Genes with ELAVL1 sites in the 3′ UTRs were similarly enriched, but genes without ELAVL1 or ZFP36 sites in their 3′ UTR did not have any enrichment. These results provide independent validation of the ZFP36 bound and regulated genes in our study using a system not reliant on ectopic overexpression of ZFP36. The enrichment of ELAVL1 gene is further support for the potential of ELAVL1 to effect expression of ZFP36 targets.

### Combinatorial regulation of mRNAs with AREs by ZFP36 and ELAVL1

**Figure 5:**
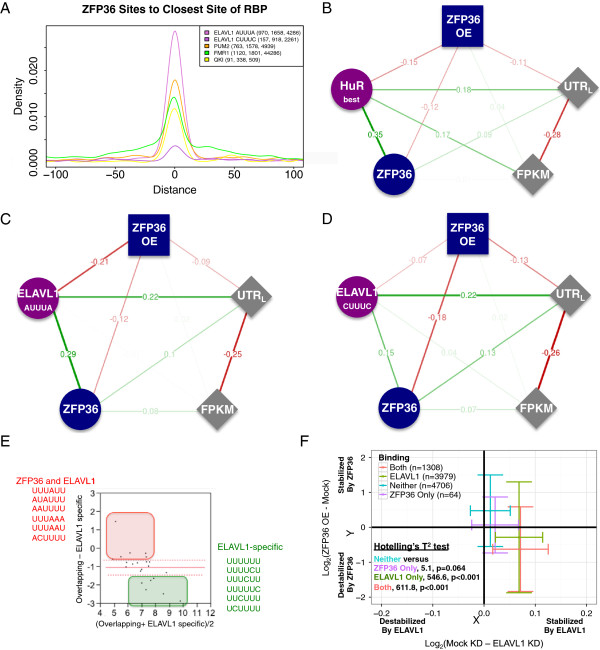
**ZFP36 and ELAVL1 combinatorial binding and regulation. (A)** In 3′ UTRs, ZFP36 sites are much closer to ELAVL1 sites containing only AUUUA (pink line) than to ELAVL1 sites containing only CUUUC (purple line). PUM2 (orange), FMR1 (green) and QKI (yellow) are included for reference. In parentheses are the number of sites in the 200-nucleotide window, the number of 3′ UTRs with sites for both ZFP36 and the RBP and the number of 3′ UTRs with sites for the RBP. **(B)** Conditionally independent correlation between the number of ZFP36 binding sites, the depth normalized number of ELAVL1_best_ binding sites and EGFP-ZFP36-induced changes in mRNA abundance. **(C, D)** ELAVL1_all_ binding sites containing AUUUA (and not UUUUU or CUUUC) correlate independently at a higher level than ELAVL1_all_ binding sites containing only CUUUC (and not UUUUU or AUUUA). **(E)** Motifs enriched in overlapping ELAVL1 and ZFP36 binding sites vs motifs enriched in ELAVL1-specific binding sites. **(F)** Scatter plot of the mean and variance of EGFP-ZFP36-induced changes in expression (on the *y*-axis, negative values indicate a reduction in EGFP-ZFP36 overexpression) and of ELAVL siRNA-knockdown-induced changes in expression (on the *x*-axis, positive values indicate a reduction in ELAVL1 knockdown) for genes with both ELAVL1 and ZFP36 sites in the 3′ UTR (red), genes with only ELAVL1 sites in the 3′ UTR (green), genes with neither ELAVL1 nor ZFP36 sites in the 3′ UTR (turquoise) and genes with only ZFP36 sites in the 3′ UTR (purple). The bars represent the variance and the point at which they cross the mean. Hotelling’s *T*^2^ score, calculated using the Hotelling R package, and Bonferroni corrected *P* values are indicated for each category. RBP, RNA-binding protein; siRNA, small interfering RNA; UTR, untranslated region.

As could be expected given the similarity in sequence preferences, there was a substantial overlap of ZFP36 and ELAVL1 sites derived by PARalyzer from PAR-CLIP data (Figure 5A and Additional file 7: Figure S4A). Specifically, over 84% of ZFP36 sites in the 3′ UTR overlapped an ELAVL1 site. Our ELAVL1 PAR-CLIP dataset corresponded well at the site level and 3′ UTR level with other ELAVL1 CLIP/PAR-CLIP data in HEK93 and HeLa cell lines (Additional file 3: Figure S1E,F). Using partial correlation analysis, we compared the independent contribution of the number of ZFP36 and ELAVL1 sites to EGFP-ZFP36-induced changes in mRNA abundance, restricted to sites in the 3′ UTR since these explained the vast majority of EGFP-ZFP36-induced changes in mRNA abundance (Figure 4E and Additional file 7: Figure S4B). After normalizing for differences in library size, the number of ELAVL1 sites and the number of ZFP36 sites both correlated to a similar extent with EGFP-ZFP36-induced changes in mRNA abundance (Figure 5B; cf. also Additional file 7: Figure S4C). Induction of EGFP-ZFP36 resulted in a 60% decrease in ELAVL1 mRNA levels. This decrease in ELAVL1 mRNA levels may explain some of the ability of ELAVL1 binding sites to explain ZFP36 overexpression changes. However, a previous study used a knockdown to reduce ELAVL1 protein levels to 15%, and reported a much smaller degree of expression changes (Additional file 4: Figure S2G) compared to the EGFP-ZFP36-induced changes in mRNA abundance [25]. Although we cannot rule out non-linear effects from the combined increase in ZFP36 concentration and decrease in ELAVL1 concentration, the data indicate that there is substantial potential for combinatorial regulation of ARE-containing mRNAs by ZFP36 and ELAVL1.

To assess specific sequences within ELAVL1-bound sites and the strength of their contribution to the EGFP-ZFP36-induced changes in mRNA abundance, we performed the same analysis using ELAVL1 sites containing CUUUC, UUUUU or AUUUA. The AUUUA ELAVL1 sites (−0.21) accounted for more EGFP-ZFP36-induced changes in mRNA abundance than the CUUUC ELAVL1 sites (−0.07) (Figure 5C,D). This result was consistent with the observation that ELAVL1 specific sites were characterized by predominantly U- and CU-rich sequences, while overlapping ELAVL1 and ZFP36 sites were characterized more by AU-rich sequences (Figure 5E). The exact same analysis using an independently derived ELAVL1 HEK293 PAR-CLIP library yielded similar results (Additional file 7: Figure S4D-F), supporting the robustness of our findings. EGFP-ZFP36-induced changes in mRNA abundance were better explained by the ARE nonamer and octamer compared to U_8_ or U_9_. ELAVL1-knockdown-responsive mRNAs had the reciprocal pattern (Additional file 6: Figure S3B). Finally, transcripts containing 3′ UTR sites for both ELAVL1 and ZFP36 had a greater concomitant decrease upon ELAVL1 knockdown and decrease upon ZFP36 overexpression, relative to transcripts containing 3′ UTR sites for only ELAVL1 or ZFP36 or no sites (Figure 5F, shifted down and to the right). Taken together, there is substantial potential for ZFP36 and ELAVL1 to compete for the same pool of binding sites, while exerting a more targeted influence on a subset of sites with factor-specific sequence preferences.

## Conclusions

Our global *in vivo* ZFP36–mRNA interaction experiments support previously reported biochemical *in vitro* ZFP36 affinity measurements, demonstrating the remarkable precision and quantitative characteristics of ZFP36 PAR-CLIP results. ZFP36 targets identified in this study were enriched for ZFP36-dependent changes in mRNA stability in mouse fibroblast cells. ZFP36 interacted with mRNAs encoding proteins important for the immune response and cancer. Many of these are other RBPs central to these two biological processes, specifically other ARE-binding RBPs such as the positive-stability regulator ELAVL1. There was extensive overlap between the interaction sites of ZFP36 and ELAVL1. Over 80% of ZFP36 sites in 3′ UTRs overlapped with ELAVL1 target sites. The preference for ZFP36 and ELAVL1 to bind and regulate mRNAs encoding RBPs suggests a complex interconnected network of post-transcriptional regulation that needs to be reconciled with respect to known ZFP36 and ELAVL1 protein expression, localization and modification dynamics, particularly in response to immune activation [54].

Partial correlation analysis accounting for confounding features demonstrated that a higher number of ZFP36 sites corresponded to stronger downregulation upon EGFP-ZFP36-induced changes in mRNA abundance. While the two proteins bind AREs, ZFP36 clearly shows a preferences towards AU- vs simply U-rich or CU-rich sequences relative to ELAVL1. This was corroborated at the level of mRNA stability, since ELAVL1 sites containing AUUUA were best able to explain EGFP-ZFP36-induced changes in mRNA abundance.

In summary, this study provides a resource for the precise transcriptome-wide RNA interactions of two ARE-binding RBPs with antagonistic functions. The precise sequence definition and function of AREs are an important question that will greatly benefit from global interaction data for these and other ARE-binding RBPs. Furthermore, we have integrated multiple genome-wide assays with computational analyses to advance our understanding of the sequence basis and extent of combinatorial gene regulation at the RNA level, in particular the ARE-mediated regulation by two of the most well-characterized RBPs.

## Materials and methods

### Cell lines

Doxycycline-inducible FLAG/HA-tagged ZFP36 HEK293 cell lines were produced utilizing the Flp-In™ T-REx™ 293 Cell Line system (Life Technologies, USA).

Flp-In™ T-REx™ HEK293 cells were generated with a FLAG/HA-tagged human ZFP36 or an EGFP-tagged human ZFP36. Both cell lines were cultured in DMEM supplemented with 10% tetracycline-reduced fetal bovine serum and appropriate selection antibiotics. For induction of FLAG/HA-tagged or EGFP-tagged ZFP36 protein, cells were treated for 16 hr with 1 μg/ml doxycycline (Sigma, USA).

### PAR-CLIP

The protocol was performed as detailed in [41] for a FLAG-HA-tagged ZFP36 and sequenced using an Illumina platform. The primary dataset comprised previously derived ELAVL1 PAR-CLIP and ELAVL1 knockdown data [25]. This was integrated with ZFP36 PAR-CLIP data. An independent ELAVL1 PAR-CLIP dataset for HEK293 cells was also used to substantiate our results. Specifically, we used the union of all ELAVL1 T1 PAR-CLIP libraries. PUM2, QKI and FMR1 HEK293 PAR-CLIP libraries were investigated to compare spatial binding biases in 3′ UTRs [55,56]. Precise analysis parameters for the PAR-CLIP libraries above are provided in Additional file 1. Binding site coordinates for ELAVL1 HEK293 CLIP and HeLa PAR-CLIP were downloaded from doRiNA [57].

Briefly, reads from the ZFP36 and ELAVL1 deep-sequencing library were stripped of the adaptor sequence using Cutadapt [58]. Reads that were less than 20 nucleotides in length or contained an ambiguous nucleotide were discarded. The remaining reads were aligned to the human genome (hg19), with up to one mismatch allowed and ten alignment locations, with Bowtie version 0.12.7 [59]. Reads with T-to-C mismatches were reconciled [42] to retain only those that mapped to a single genomic location for the minimum number of mismatches. Annotation was performed as described in [56]. Full site- and gene-level data for the primary data compared are in Additional file 2: Table S1 and Additional file 5: Table S2, respectively.

### Motif affinity

The SNR values were calculated for each probe sequence in Table one in [48] appearing at least once in 3′ UTR ZFP36 binding sites. The SNR was calculated as in [42], and is defined as the number of instances per nucleotide in a given set of PARalyzer interaction sites, divided by the number of instances per nucleotide in the background set. In this case the background was the sequence of the longest annotated 3′ UTR for a gene. The SNR values were plotted against the *K* and *K*_*d*_ values from [48]. The formula for the power curve fit of the SNR to *K*_*d*_ was used to predict *K*_*d*_ values for other sequences.

### Microarray samples and analysis

EGFP-ZFP36 cells were passaged at 1:10 dilution in 15 ml of media in ten T75 flasks. They were allowed to grow for three days to 60% confluence. They were then treated with doxycycline (Sigma D989) to a final concentration of 1 μg/ml. The same volume of sterile water was used as a control. The cells were incubated for 16 hr at 37°C, in 5% CO_2_. The flasks without doxycycline were about 80% confluent. Cells with doxycycline were about 40% confluent and there were more floating cells and rounded cells. The cells were harvested by trypsinizing. Cells from five flasks per treatment were pooled. They were centrifuged at 500 *g* for 5 min, and resuspended in growth medium to final concentrations of 1 × 10^7^ cells/ml. Fluorescence-activated cell sorting (FACS) was done using a FACSAria II from Becton Dickinson (San Jose, CA). Cells were excited at 488 nm and sorted based on their GFP fluorescence at 525/550 nm.

The FacsDiva software version 6.1.3 was used for analysis. Doublet discrimination gates were set on forward scatter and side scatter to ensure the sorting of single cells. The sort gate was set on a histogram of GFP + cells for a fluorescence signal of greater than 10^3^ log units. Control cells were sorted in the same way, except that fluorescent cells were excluded (Additional file 3: Figure S1B). The sorted cells were collected into DMEM with 10% FBS in 6 ml polypropylene tubes at approximately 2 × 10^6^ cells per tube. They were pelleted at 500 *g* for 20 min at 4°C and washed in PBS, then transferred into 1.5 microfuge tubes at approximately 4 × 10^6^ cells per tube. These were centrifuged at 500*g* for 20 to 30 min at 4°C. The supernatant was removed, and the pellets were snap frozen in liquid nitrogen and stored at −80°C. This experiment was repeated on five independent days, using cells from passages 17, 20, 23, 26 and 27. RNA was isolated from approximately 3 × 10^6^ to 6 × 10^6^ cells using the GE Illustra RNAspin mini kit. To control for doxycycline effects, parental HEK293 cells were passaged at 1:4 or 1:5 dilution in 15 ml medium in four T75 flasks. They grew for 2 days to approximately 60% confluence. They were then treated with doxycycline in the same way, that is, 1 μg/ml for 16 hr at 37°C in 5% CO_2_. Control cells were treated with the same volume of sterile water. The cells were harvested by trypsinizing, and processed and used for the microarray in the same way as for the GFP + cells. The same experiment was performed on five different days, and comparisons were made using five doxycycline treated vs five vehicle (water) treated samples, with each experiment done as pairs of treated and untreated cells. The samples were stored at −80°C. The integrity of the RNA was confirmed by running 3 μg of each RNA sample on 1.2% agarose/formaldehyde gels.

Then 1.5 μg of RNA from each sample was provided to the NIEHS Microarray Core Facility for analysis, using an Affymetrix Human Genome U133 Plus 2.0 gene chip (Affymetrix, Santa Clara, CA). As directed in the Affymetrix 3′ IVT Express kit protocol, 100 ng of total RNA was amplified. Then 12.5 μg of amplified biotin-RNA was fragmented and 10 μg were hybridized to each array for 16 hr at 45°C in a rotating hybridization oven using the Affymetrix Eukaryotic Target Hybridization Controls and protocol. Array slides were stained with streptavidin/phycoerythrin utilizing a double-antibody staining procedure and then washed for antibody amplification according to the GeneChip Hybridization, Wash and Stain Kit and user manual. Arrays were scanned in an Affymetrix Scanner 3000 and data were obtained using the GeneChip® Command Console Software (AGCC; Version 1.1). The data were normalized using gcrma [60] and then filtered to retain only unambiguously mapping probes and minimal expression level (mean log hybridization value for replicates within a group > 4.5). Bonferroni corrected *P* values for fold changes were calculated using Gene Pattern [61]. Gene set enrichment analysis [62] was used to compare HEK293 data to ZFP36 mouse fibroblast mRNA stability data. Human–mouse mappings were done using an appropriate microarray chip. The probes were ranked by differential mRNA half-life score, with higher scores indicating a larger slope in ZFP36 KO than ZFP36 WT cells (for details refer to [53]).

### Partial correlation

Partial correlation analysis was performed using the R package pcor.R with Spearman correlations coefficients computed by the recursive formula [35]. Partial correlation analysis measures the degree of association between tworandom variables, independent of some set of other random variables. Formally, the partial correlation, *ρXY***Z**, is defined as the correlation between the residuals, *R*_*X*_ and *R*_*Y*_, found using linear regression of *X* with *Z* and *Y* with *Z* where *Z* is the set of variables for which we are controlling.

$$\begin{array}{l} w_{X}^{*}=\arg\min_{w}\left\{\sum_{i=1}^{N}{\left(x_{i}-\langle w,z_{i}\rangle \right)}^{2}\right\}\;\mathrm{and}\\ w_{Y}^{*}=\arg\min_{w}\left\{\sum_{i=1}^{N}{\left(y_{i}-\langle w,z_{i}\rangle \right)}^{2}\right\} \end{array}$$

*N* is the number of variables in the set *Z* and 〈**w**, **z**_**i**_〉 is the scalar product of the vectors **w** and **z**_**i**_. The residuals can then be written as:

$$\begin{array}{c} r_{X,i}=x_{i}-\langle w_{X}^{*},z_{i}\rangle \\ r_{Y,i}=y_{i}-\langle w_{Y}^{*},z_{i}\rangle \end{array}$$

From here, the partial correlation can be obtained using the formula:

$$\hat{\rho }\mathrm{XY}\;Z=\frac{N\sum_{i=1}^{N}r_{X,i}r_{Y,i}-\sum_{i=1}^{N}r_{X,i}\sum_{i=1}^{N}r_{Y,i}}{\sqrt{N\sum_{i=1}^{N}r_{x,i}^{2}-{\left(\sum_{i-1}^{N}r_{X,i}\right)}^{2}}\sqrt{N\sum_{i=1}^{N}r_{Y,i}^{2}-{\left(\sum_{i-1}^{N}r_{Y,i}\right)}^{2}}}$$

It is common to use a recursive algorithm utilizing the reducibility of this *n*th-order formula to three (*n* – 1)th-order partial correlations, represented by the formula:

$$\mathrm{\rho XY}\;Z=\frac{\mathrm{\rho XY}\;Z\backslash \left\{Z_{0}\right\}-\mathrm{\rho X}Y_{0}\;Z\backslash \left\{Z_{0}\right\}\rho Z_{0}Y\;Z\backslash \left\{Z_{0}\right\}}{\sqrt{1-\rho_{X\;Z_{0}\;Z\backslash \left\{Z_{0}\right\}}^{2}}\sqrt{1-\rho_{Z_{0}\;Y\;Z\backslash \left\{Z_{0}\right\}}^{2}}}$$

**Figure 6:**
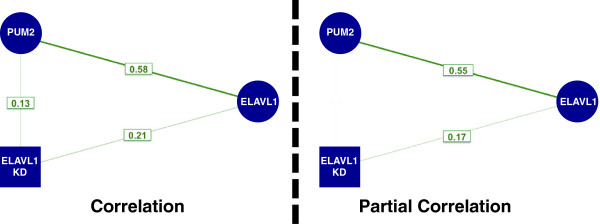
**Correlation vs partial correlation example.** Correlation (left) and partial correlation (right) analyses of number PUM2 sites per 3′ UTR (blue circle), ELAVL1 3′ UTR sites and ELAVL1-siRNA-induced changes in mRNA abundance (blue square). siRNA, small interfering RNA; UTR, untranslated region.

*X* is the number of ELAVL 3′ UTR binding sites, *Y* is the number of PUM2 3′ UTR binding sites and *Z* is the log_2_ change in expression due to HuR knockdown. The correlation analysis between these three variables (Figure 6, left) shows there is a positive relation between all three variables, which could lead one to believe that PUM2 binding sites could explain changes in gene expression upon HuR knockdown. However, the partial correlation or the correlation between the residuals, *R*_*X*_ and *R*_*Y*_ (Figure 6, right), reveals that PUM2 binding sites do not explain any additional changes in gene expression upon HuR knockdown once HuR binding sites have been accounted for. Accounting for such interdependencies is clearly important for interpreting regulatory mechanisms when integrating genomic datasets.

### Analysis of spatial patterns

To visualize binding along transcripts, cluster data was taken from PARalyzer output and annotated. Only 3′ UTR, 5′ UTR, coding and intron annotation categories were utilized. The transcript segment associated with the annotation category of each cluster was selected using a GENCODE version 18 gene transfer format file [63]. For each gene, we used the transcript that had the longest segment matching the binding site of the cluster. The location and relevant distance along the transcript segment were then calculated. For each of the relevant annotation categories, a density plot was created utilizing each of these distances. Permutations of the observed data were used to create the background data distributions. For each cluster we created a random site along the length of its associated transcript segment repeated 100 times. The cluster plot function in the R LSD package [64], was used to plot the observed and permuted data. The LSD cluster plot function creates a graph in which the black center line is the median, and the thick gray lines separating the color gradients around the black line represent the upper and lower quartiles. The color gradient continues until the 10th and 90th percentiles.

### Data availability

Both ZFP36 PAR-CLIP sequencing and overexpression microarray datasets have been deposited into GEO [GEO:GSE53185].

## Abbreviations

ARE: AU-rich element; DMEM: Dulbecco’s modified Eagle’s medium; GFP: green fluorescent protein; KO: knockout; miRNA: microRNA; ncRNA: non-coding RNA; NMR: nuclear magnetic resonance; PAR-CLIP: photoactivatable ribonucleoside cross-linking and immunoprecipitation; PBS: phosphate-buffered saline; RBP: RNA-binding protein; RNP: ribonucleoprotein; RRE: RNA regulatory element; siRNA: small interfering RNA; SNR: signal-to-noise ratio; TNF: tumor necrosis factor; TZF: tandem CCCH zinc finger; UTR: untranslated region; WT: wild type.

## Competing interests

TT is a cofounder of and scientific advisor to Alnylam Pharmaceuticals and a scientific advisor to Regulus Therapeutics.

## Authors’ contributions

NM, PJB and UO conceived and designed the experiments. PJB and TT designed the ZFP36 PAR-CLIP protocol. MH and JDN performed the PAR-CLIP. EAK and PJB designed and carried out the ZFP36 overexpression experiments. NM and NCJ performed the computational analyses. NM and UO wrote the manuscript with input from NCJ, PJB, MH and TT. All authors read and approved the final manuscript.

## Supplementary Material

Additional file 1 **Analysis parameters.** Precise analysis parameter files for ZFP36 and ELAVL1 PAR-CLIP libraries. Click here for file

Additional file 2: Table S1 Comma-separated values of ZFP36 PAR-CLIP binding sites. Click here for file

Additional file 3: Figure S1 ZFP36 and ELAVL1 PAR-CLIP library statistics. **(A)** Processing and summary statistics for reads and sites identified by ZFP36 PAR-CLIP. **(B)** Bubble plot comparing number of ZFP36 sites, the fraction of reads with a T-to-C conversion per cluster, and conversion specificity, which is log_10_(#T-to-C reads/(1 + # reads with conversions not T-to-C)). **(C)** Number of sites in mRNA regions for primary libraries utilized (ZFP36 and ELAVL1). **(D)** Distribution of PAR-CLIP binding sites in length-normalized 3′ UTRs for RBPs indicated by color with the counts in parentheses. **(E)** Distance to other ELAVL1 3′ UTR sites. (P) indicates the binding site coordinates were generated by our pipeline and (d) indicates the binding site coordinates were downloaded from doRiNA. PUM2 PAR-CLIP data, shown as a reference, from HEK293 cells. For each library the numbers in parentheses are the number of sites utilized in the plot, the total number of sites annotated as 3′ UTRs and the total number of genes containing at least one 3′ UTR site. **(F)** Venn diagram of the overlap of genes with 3′ UTRs with at least one binding site for the libraries indicated. Primary ELAVL1 was the dataset used throughout the current study. Click here for file

Additional file 4: Figure S2 ZFP36 overexpression analysis. **(A)** Western blot probed with monoclonal ZFP36 antibodies demonstrating doxycycline-induced EGFP-ZFP36 expression (left two lanes) and transfection of pBluescript (BS+) or ZFP36 cDNA plasmid into HEK293 cells (right two lanes). **(B)** Fluorescence-activated cell sorting (FACS) analysis of EGFP-ZFP36 expression treated with vehicle (above) or doxycycline (below) see Materials and methods for details. **(C)** Distribution of log_2_ fold change (left) and Bonferroni corrected *P* values (right) for ZFP36 vs mock and doxycycline vs vehicle. **(D)** log_2_ fold change distribution of significantly differentially expressed genes (*P* < 0.01). **(E)** Top enriched motifs identified by miREDUCE analysis of ZFP36 overexpression. **(F)** Correlation between motif occurrence in 3′ UTR and ZFP36 overexpression. **(G)** log_2_ fold change distribution for mock knockdown vs ELAVL1 knockdown. **(H)** Comparison between 3′ UTRs of transcripts significantly downregulated or upregulated upon ZFP36 overexpression by categories defined by PAR-CLIP sites with specific attributes indicated. Click here for file

Additional file 5: Table S2 Comma-separated values of ZFP36 overexpression data and ZFP36 PAR-CLIP binding sites summarized at the gene level. Click here for file

Additional file 6: Figure S3 Comparison of ELAVL1 and ZFP36 targets for ZFP36-deficient mRNA half-life data and known motifs. **(A)** Gene set enrichment analysis (GSEA) results for mouse probes ranked by score reflecting the differences in mRNA half-life between serum-stimulated ZFP36 KO and WT mouse fibroblasts. Name of gene set indicates data utilized, which included ZFP36 RNP immunoprecipitation-microarray (RIP-chip) targets from activated mouse macrophage RAW cells and ELAVL1 RIP-chip targets from a human Jurkat activation time course [18,65]. The size, normalized enrichment score (NES) and multiple hypothesis corrected significance value (FWER *P*-val) are indicated. Colored bars within each column indicate the relative value. **(B)** Classical ZFP36-targeted nonamers and octamers correlate more strongly with ZFP36 overexpression than strings of Us of equivalent length. The opposite relation was observed for ELAVL1 knockdown. **(C)** Gene ontology enrichment analysis of genes with ZFP36 binding sites ranked by Bonferroni corrected *P* values (< 0.05) from the Panther DB molecular function category using the the differences in mRNA half-life scores as expression ranks. Click here for file

Additional file 7: Figure S4 Further relations for ELAVL1 and ZFP36 overexpression. **(A)** ZFP36 sites are closer to their nearest ELAVL1 sites (blue line) than background simulations. **(B)** ELAVL1 binding sites in the 3′ UTR are far more highly correlated with ZFP36 overexpression than those in the 5′ UTR, coding region or intron. **(C)** The number of ELAVL1_all_ binding sites correlates independently with ZFP36 overexpression at a level much greater than the number of ZFP36 binding sites. Panels D, E and F utilize independently derived HEK293 ELAVL1 PAR-CLIP data from [26], which is colored a lighter purple. **(D)** The number of ELAVL1 binding sites found by Kishore *et al*. correlates independently with ZFP36 overexpression at a level much greater than the number of ZFP36 binding sites. **(E, F)** ELAVL1 binding sites found by Kishore *et al*. containing AUUUA (and not UUUUU or CUUUC) correlate independently at a higher level than ELAVL1 binding sites found by Kishroe *et al*. containing only CUUUC (and not UUUUU or AUUUA). Click here for file
