# Supplementary material for: Global target mRNA specification and regulation by the RNA-binding protein ZFP36
Source: Genome Biol. 2014 Jan 8;15(1):R12. doi: 10.1186/gb-2014-15-1-r12 (PMC4053807; doi:10.1186/gb-2014-15-1-r12)

Supplemental Figure 1

A)

Summary Stats	ZFP36	Percent
Reads	6361864	100
Filtered Total	3405506	53.53
Quality	19208	0.3
Length <20 nts	3386298	53.23
3' Adapter	5890211	92.59
5' Adapter	71622	1.13
19 nt Marker	17690	0.28
24 nt Marker	20682	0.33
Post-Processing	2956358	46.47
Uniquely Aligned Reconciled Reads	328433	11.11
Site	4626	

Annotation	Reads	T2C Fraction	Conv Spec	Sites
3'utr	35011	0.82	1.34	2314
5'utr	1056	0.38	0.01	20
coding	7041	0.28	0.02	84
NoAnnotation	63021	0.33	-0.18	635
intron	38745	0.61	0.41	871
lincRNA	3387	0.4	0	84
miRNA	1936	0.03	-0.83	6
miscRNA	57	0.12	-0.11	0
Mt rRNA	605	0.15	0.07	2
Mt tRNA	130	0.04	-0.94	1
piRNA	14	0.07	-0.78	3
repeat	29970	0.51	0.18	494
rRNA	143782	0.13	-0.32	94
snoRNA	2502	0.03	-0.63	6
snRNA	270	0.21	0.2	4
tRNA	906	0.09	-0.74	8

B)

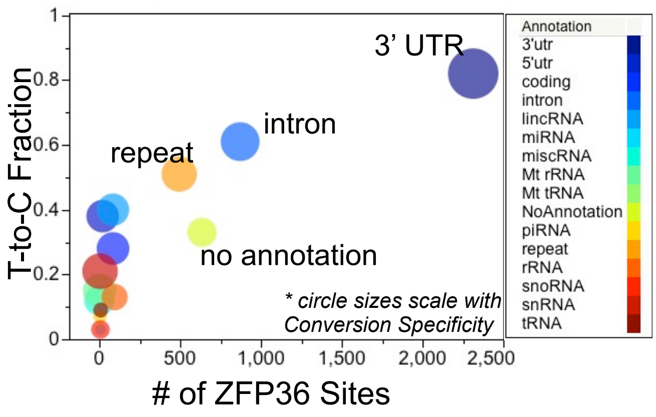

C)

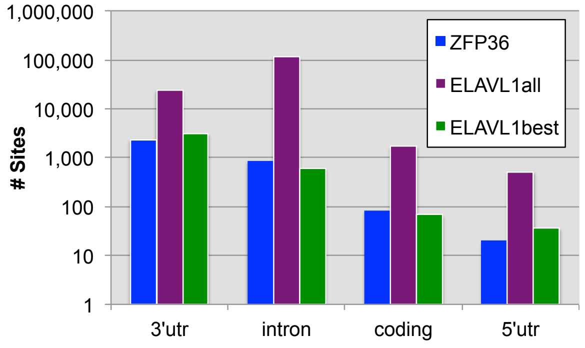

D)

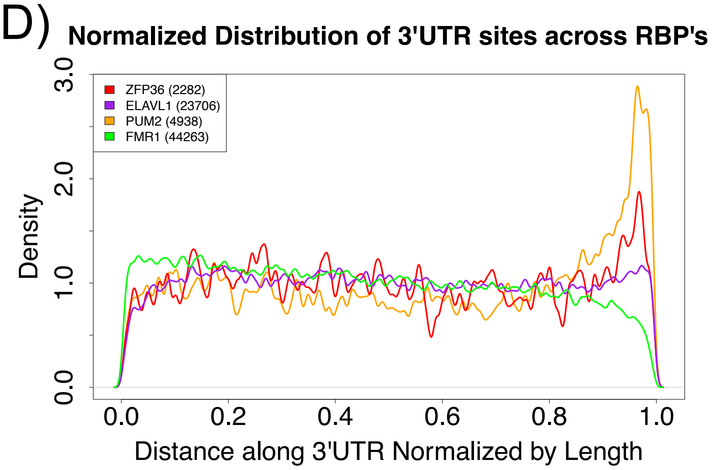

E)

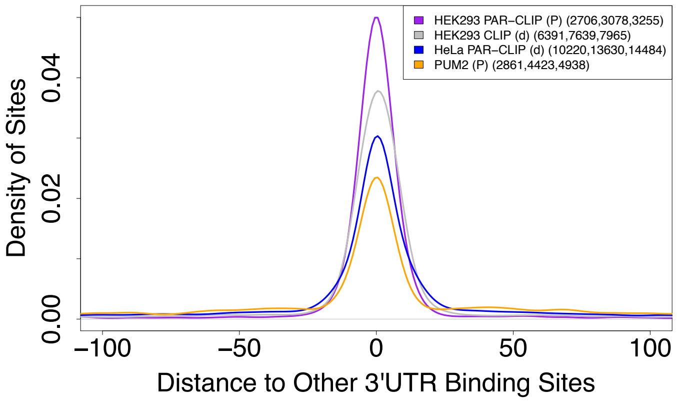

F)

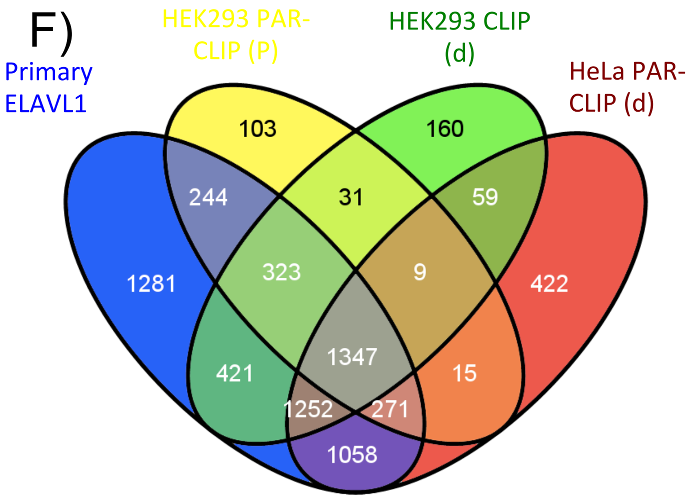

Supplement: Additional file 3: Figure S1 — ZFP36 and ELAVL1 PAR-CLIP library statistics. (A) Processing and summary statistics for reads and sites identified by ZFP36 PAR-CLIP. (B) Bubble plot comparing number of ZFP36 sites, the fraction of reads with a T-to-C conversion per cluster, and conversion specificity, which is log10(#T-to-C reads/(1 + # reads with conversions not T-to-C)). (C) Number of sites in mRNA regions for primary libraries utilized (ZFP36 and ELAVL1). (D) Distribution of PAR-CLIP binding sites in length-normalized 3′ UTRs for RBPs indicated by color with the counts in parentheses. (E) Distance to other ELAVL1 3′ UTR sites. (P) indicates the binding site coordinates were generated by our pipeline and (d) indicates the binding site coordinates were downloaded from doRiNA. PUM2 PAR-CLIP data, shown as a reference, from HEK293 cells. For each library the numbers in parentheses are the number of sites utilized in the plot, the total number of sites annotated as 3′ UTRs and the total number of genes containing at least one 3′ UTR site. (F) Venn diagram of the overlap of genes with 3′ UTRs with at least one binding site for the libraries indicated. Primary ELAVL1 was the dataset used throughout the current study. [file gb-2014-15-1-r12-S3.pdf]
