# Supplementary material for: Global target mRNA specification and regulation by the RNA-binding protein ZFP36
Source: Genome Biol. 2014 Jan 8;15(1):R12. doi: 10.1186/gb-2014-15-1-r12 (PMC4053807; doi:10.1186/gb-2014-15-1-r12)
Supplement: Additional file 4: Figure S2 — ZFP36 overexpression analysis. (A) Western blot probed with monoclonal ZFP36 antibodies demonstrating doxycycline-induced EGFP-ZFP36 expression (left two lanes) and transfection of pBluescript (BS+) or ZFP36 cDNA plasmid into HEK293 cells (right two lanes). (B) Fluorescence-activated cell sorting (FACS) analysis of EGFP-ZFP36 expression treated with vehicle (above) or doxycycline (below) see Materials and methods for details. (C) Distribution of log2 fold change (left) and Bonferroni corrected P values (right) for ZFP36 vs mock and doxycycline vs vehicle. (D) log2 fold change distribution of significantly differentially expressed genes (P < 0.01). (E) Top enriched motifs identified by miREDUCE analysis of ZFP36 overexpression. (F) Correlation between motif occurrence in 3′ UTR and ZFP36 overexpression. (G) log2 fold change distribution for mock knockdown vs ELAVL1 knockdown. (H) Comparison between 3′ UTRs of transcripts significantly downregulated or upregulated upon ZFP36 overexpression by categories defined by PAR-CLIP sites with specific attributes indicated. [file gb-2014-15-1-r12-S4.pdf]

Supplemental Figure 2

A)

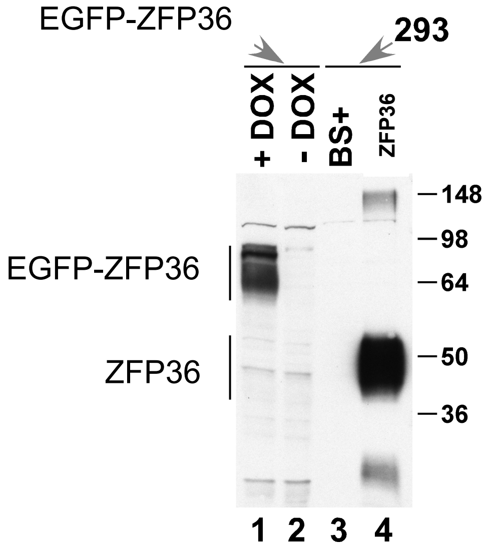

B)

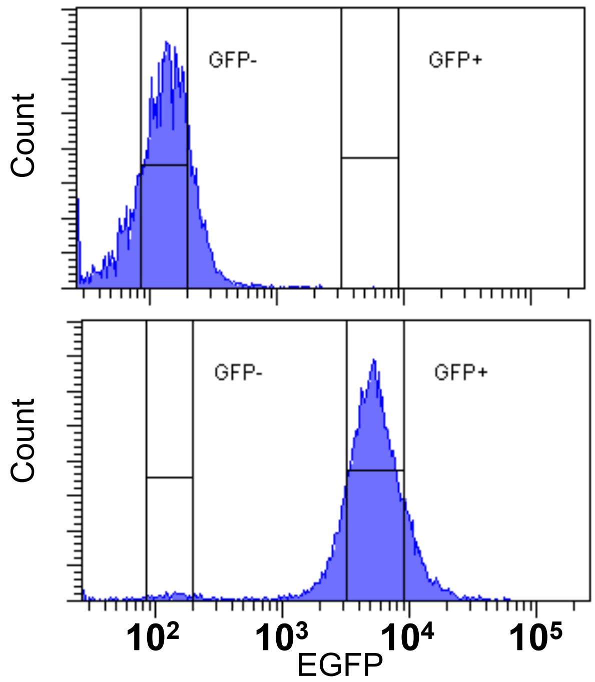

C)

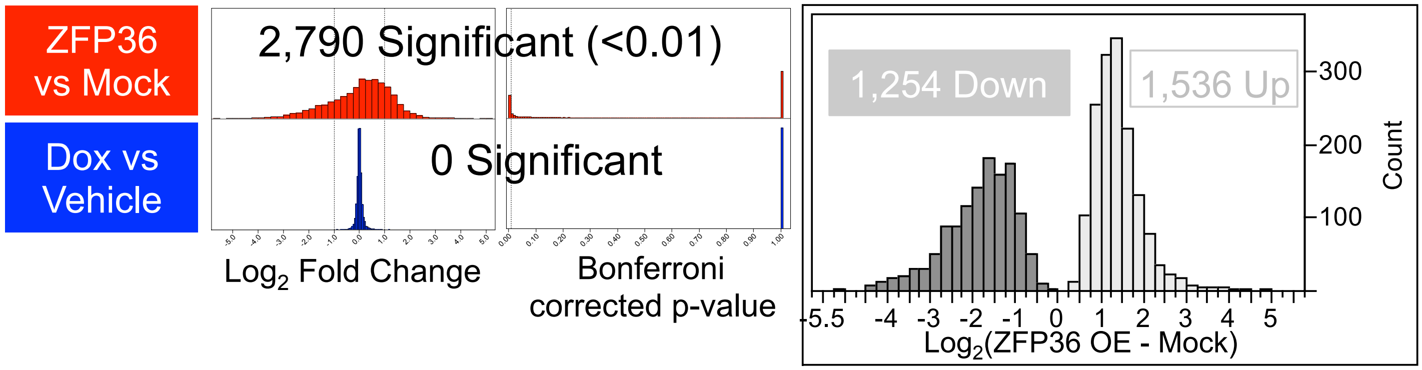

E)

motif	regr_coef	p_val	no_of_genes	avg_LFCr
ATATTTA	-0.104	0	3187	-0.553
TATTTTA	-0.102	0	4108	-0.482
GTATTTA	-0.09	0	2381	-0.548
TATTATT	-0.072	1.85E-08	2593	-0.57
TAATATT	-0.07	0.007693	2446	-0.614
CTATTTA	-0.064	5.45E-10	1854	-0.482
TGATTTA	-0.062	5.99E-08	1972	-0.497
TATTAAT	-0.061	0	2126	-0.581
TTATGTA	-0.057	1.03E-05	2080	-0.551
TTAAGAT	-0.053	0.002227	1684	-0.529
GTAATTA	-0.048	0.0007705	1328	-0.541
TAACTAT	-0.043	0.04274	1110	-0.624

F)

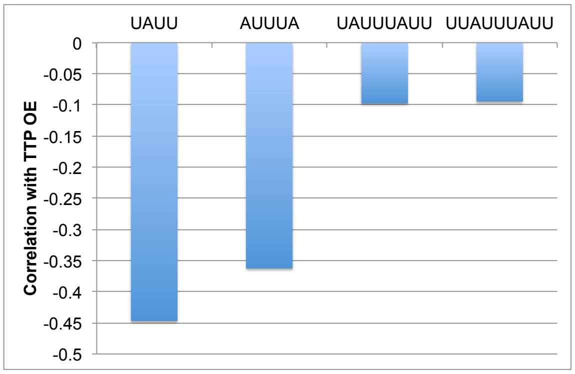

G)

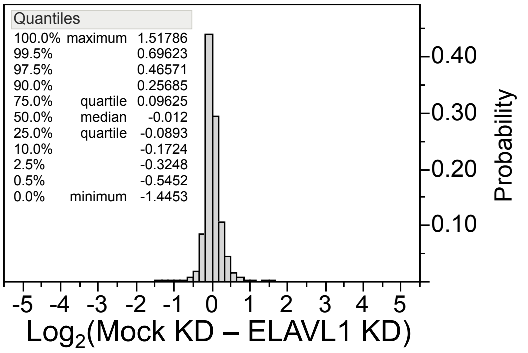

I)

Category	Downregulated		Upregulated	
	#	%	#	%
ZFP36 Only	3	0.24%	4	0.26%
ELAVL1 Only	718	57.26%	485	31.58%
Both	314	25.04%	62	4.04%
ZFP36 UAUU	285	22.73%	59	3.84%
ZFP36 AUUUA	168	13.40%	33	2.15%
ZFP36 UAUUUAUU	36	2.87%	11	0.72%
ZFP36 UUAUUUAUU	15	1.20%	3	0.20%
ELAVL1 UAUU	906	72.25%	306	19.92%
ELAVL1 AUUUA	614	48.96%	134	8.72%
ELAVL1 UAUUUAUU	103	8.21%	15	0.98%
ELAVL1 UUAUUUAUU	44	3.51%	6	0.39%
Total	1254	100.00%	1536	100.00%
