# Supplementary material for: Global target mRNA specification and regulation by the RNA-binding protein ZFP36
Source: Genome Biol. 2014 Jan 8;15(1):R12. doi: 10.1186/gb-2014-15-1-r12 (PMC4053807; doi:10.1186/gb-2014-15-1-r12)
Supplement: Additional file 6: Figure S3 — Comparison of ELAVL1 and ZFP36 targets for ZFP36-deficient mRNA half-life data and known motifs. (A) Gene set enrichment analysis (GSEA) results for mouse probes ranked by score reflecting the differences in mRNA half-life between serum-stimulated ZFP36 KO and WT mouse fibroblasts. Name of gene set indicates data utilized, which included ZFP36 RNP immunoprecipitation-microarray (RIP-chip) targets from activated mouse macrophage RAW cells and ELAVL1 RIP-chip targets from a human Jurkat activation time course [18,65]. The size, normalized enrichment score (NES) and multiple hypothesis corrected significance value (FWER P-val) are indicated. Colored bars within each column indicate the relative value. (B) Classical ZFP36-targeted nonamers and octamers correlate more strongly with ZFP36 overexpression than strings of Us of equivalent length. The opposite relation was observed for ELAVL1 knockdown. (C) Gene ontology enrichment analysis of genes with ZFP36 binding sites ranked by Bonferroni corrected P values (< 0.05) from the Panther DB molecular function category using the the differences in mRNA half-life scores as expression ranks. [file gb-2014-15-1-r12-S6.pdf]

Supplemental Figure 3

A)

Gene Set	SIZE	NES	FWER p-val
ELAVL1 ALL JURKAT RIP	543	1.99	0
ZFP36 OE DOWN	927	1.8	0.001
ELAVL1 ONLY PAR	3290	1.77	0.001
ZFP36 OE DOWN AND PAR	234	1.74	0.002
ZFP36 AND ELAVL1 PAR	1036	1.73	0.002
ZFP36 PAR	1110	1.71	0.003
ZFP36 OE UP AND PAR	105	1.63	0.012
ZFP36 OE UP	1058	1.45	0.1
ZFP36 ONLY PAR	59	1.29	0.437
ZFP36 RAW	88	1.01	1

B)

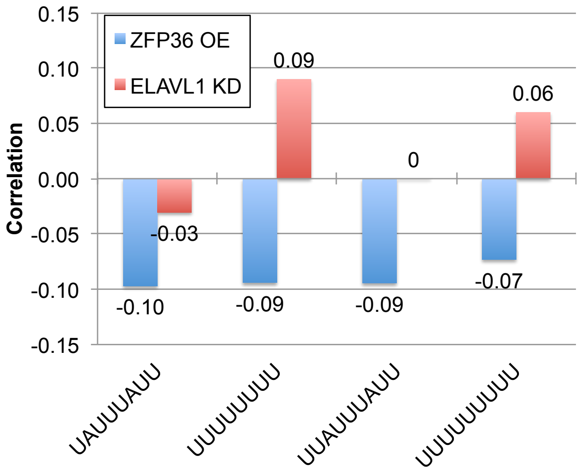

C)

Molecular Function	Size	Enrichment p-value
RNA binding	365	1.51E-06
mRNA binding	214	2.26E-05
RNA splicing factor activity, transesterification mechanism	175	4.75E-05
protein kinase activity	431	9.27E-04
ubiquitin-protein ligase activity	189	3.03E-03
transferase activity	1318	3.29E-03
ligase activity	384	6.32E-03
kinase activity	561	1.18E-02
translation initiation factor activity	56	1.91E-02
catalytic activity	4165	3.47E-02
