# Supplementary figures and images for: Global target mRNA specification and regulation by the RNA-binding protein ZFP36
Source: Genome Biol. 2014 Jan 8;15(1):R12. doi: 10.1186/gb-2014-15-1-r12 (PMC4053807; doi:10.1186/gb-2014-15-1-r12)

# Supplemental Figure 4

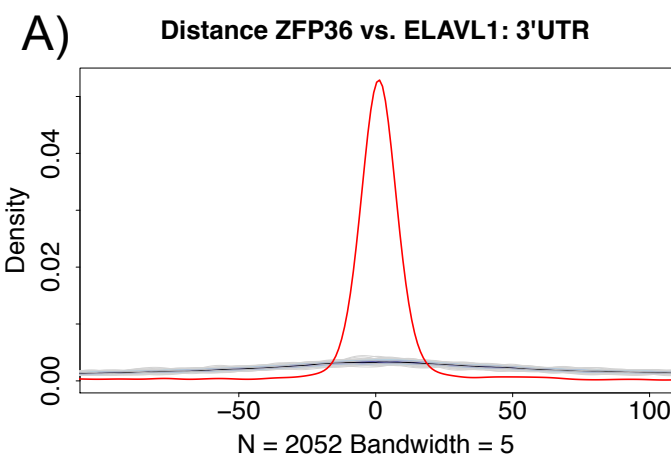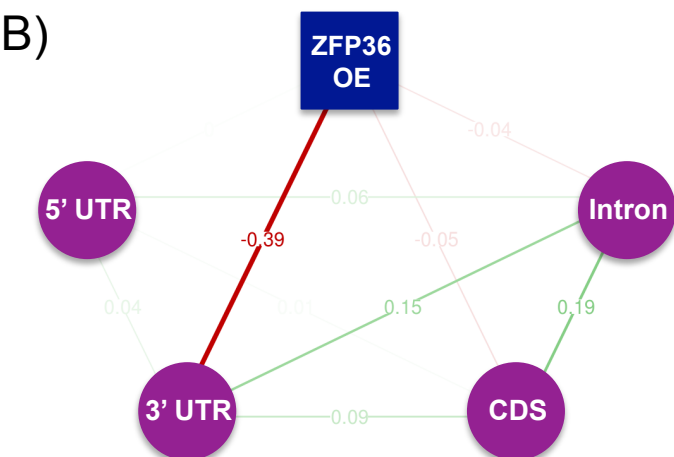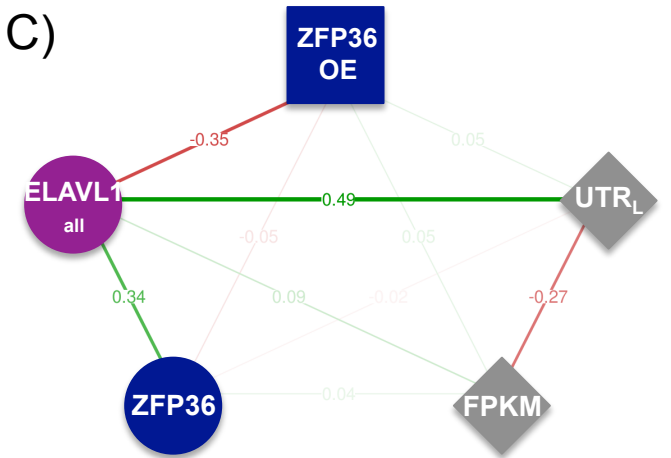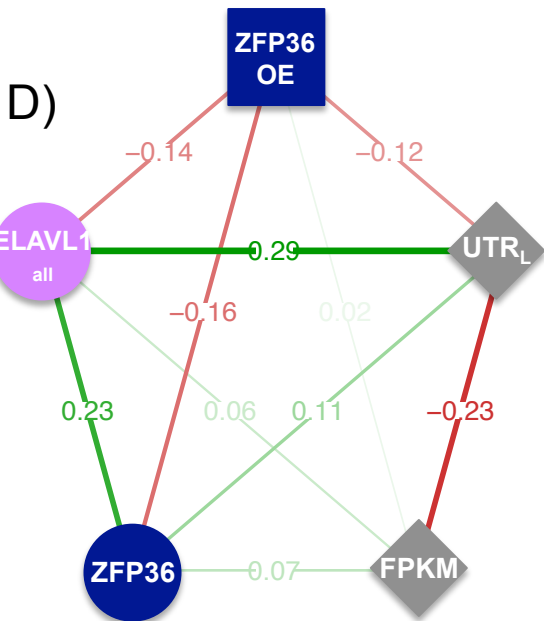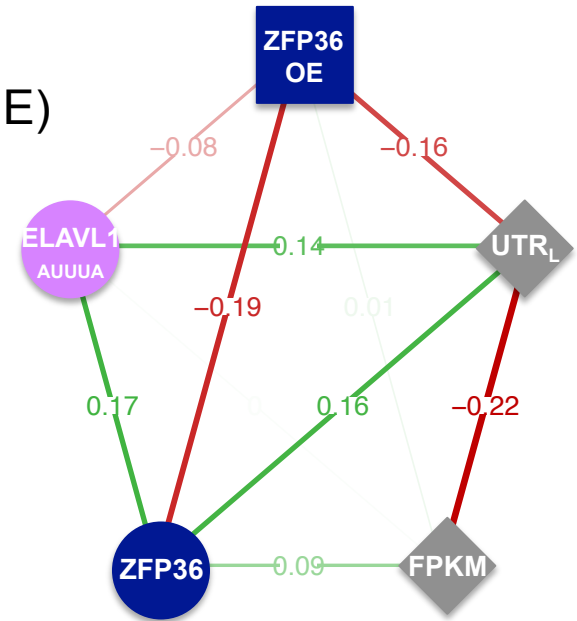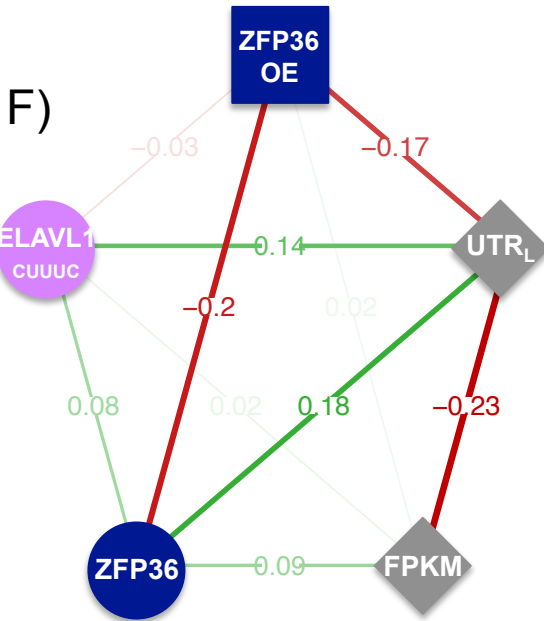

Supplement: Additional file 7: Figure S4 — Further relations for ELAVL1 and ZFP36 overexpression. (A) ZFP36 sites are closer to their nearest ELAVL1 sites (blue line) than background simulations. (B) ELAVL1 binding sites in the 3′ UTR are far more highly correlated with ZFP36 overexpression than those in the 5′ UTR, coding region or intron. (C) The number of ELAVL1all binding sites correlates independently with ZFP36 overexpression at a level much greater than the number of ZFP36 binding sites. Panels D, E and F utilize independently derived HEK293 ELAVL1 PAR-CLIP data from [26], which is colored a lighter purple. (D) The number of ELAVL1 binding sites found by Kishore et al. correlates independently with ZFP36 overexpression at a level much greater than the number of ZFP36 binding sites. (E, F) ELAVL1 binding sites found by Kishore et al. containing AUUUA (and not UUUUU or CUUUC) correlate independently at a higher level than ELAVL1 binding sites found by Kishroe et al. containing only CUUUC (and not UUUUU or AUUUA). [file gb-2014-15-1-r12-S7.pdf]
